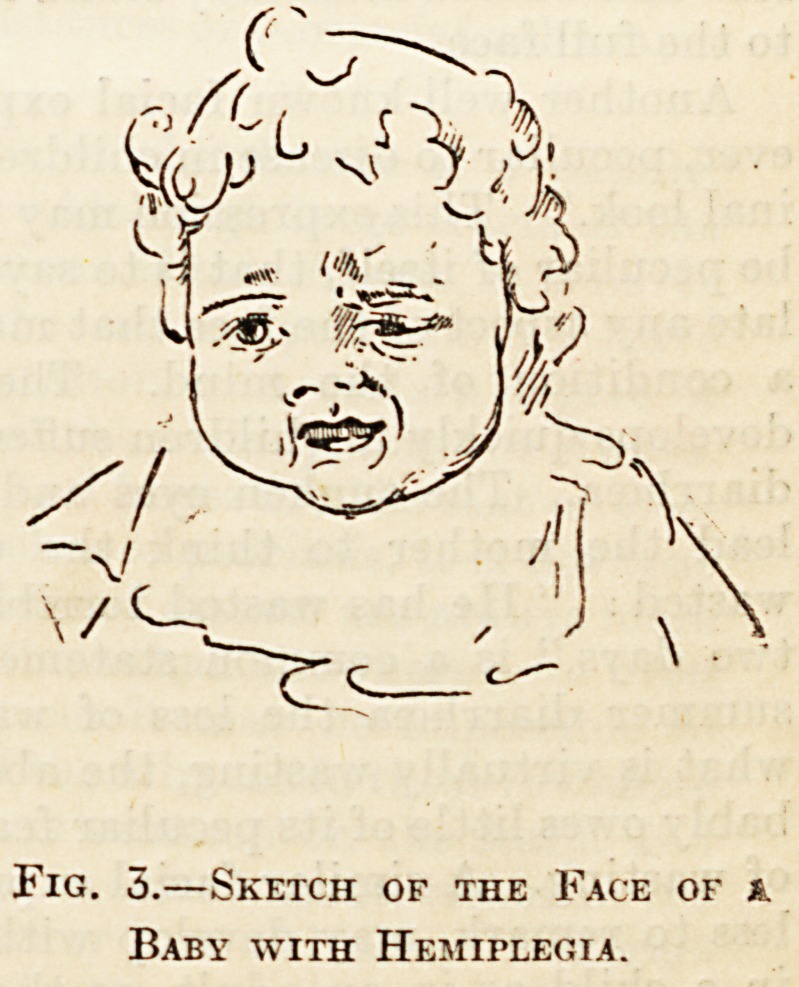# The Facial Aspect in Diseases of Children

**Published:** 1906-04-07

**Authors:** Theodore Fisher

**Affiliations:** Assistant Physician to the East London Hospital for Children


### THE FACIAL ASPECT IN DISEASES OF CHILDREN.

By THEODORE FISHER, M.D., M.R.C.P., Assistant Physician to the East London Hospital for Children.

It has been said that "the most interesting study to man is man." We are always consciously or unconsciously studying those around us, and it is the expression of the face that affords us most opportunity of learning something of the nature that lies behind it. Just as a face to a great extent indicates character, so also it shows, more or less distinctly, varied conditions of health, and in a lesser degree may reveal the nature of a complaint from which anyone may be suffering.

The nature of disease is perhaps more readily discerned in the faces of children than in those of adults. The normal child, at least the very young child, may be said to be free from those disturbing impulses which come from above, that is, from the brain and mind, which often mark the faces of their elders with an expression of pain. The face of the true child is one of restful happiness, and when an aspect of fixed distress appears, it indicates, not painful working of the mind, but discomfort or disease. To this there are exceptions; for example, in some mentally defective children ordinary processes of thought appear to require painful effort, the evidence of which is seen in puckering of the forehead. Of the general truth, however, of the observation that, in at least a young child, happiness depends upon internal comfort, we have only to think of a baby whose food agrees with it, and of one less fortunately circumstanced. At this period of life temper generally means colic, and smiles, smoothly working digestive organs.

The expression, however, in sick children is not by any means one always distinctly suggestive of pain. It has already been mentioned that expressions dependent mainly upon states of the mind are not to be expected in children. Yet, although this is true, the expressions produced by disease are often strongly suggestive rather of some form of disquiet of the mind than of distress originating in the body. There may be, for example, an expression of distressed anxiety, of fear, or of philosophic disregard for the trifles of life.

To take the first, the expression of thoughtful anxiety, this may be present comparatively early in cases of meningitis. Thus, Dr. John Thomson gives an illustration from a photograph of a case of meningitis in a child, and comments upon it as follows. He says: "the eyes are closed, he is knitting his brows . . . the central irritation is producing tight contraction of the masseters, . . . the general aspect of the upper half of the face is that of deep and earnest thought, while the clenched jaw, and slight drawing back of the head give the impression of stern determination." I have added an illustration from a note-book of my own. The sketch was made and the accompanying notes jotted down before I had seen the remarks of Dr. Thomson and others upon the thoughtful expression of meningitis. My own notes are as follow: "The eyes shut, pained expression of face, contraction of the muscles of forehead, around mouth, and of the platysma, cannot open the mouth more than half an inch." In the mention of the frowning forehead, closed eyes, and clenched jaw, the description of my case resembles that of Dr. Thomson's, but Dr. Thomson describes the expression in his case as that of "deep and earnest thought," while I speak of a "pained expression." I think, however, my sketch is perhaps as suggestive of thought as of pain.

FIG. 1.—SKETCH OF A CASE OF MENINGITIS.

FIG. 2.—SKETCH OF A CASE OF ASTHMA.

FIG. 3.—SKETCH OF THE FACE OF A BABY WITH HEMIPLEGIA.

Deep and earnest thought is not, however, the only form of thought suggested by the expression of disease in children. In some cases it is one of philosophic melancholy. In neglected cases of empyema, for example, and also in some cases of chronic lung disease, such as dilatation of the bronchial tubes, sadness is the prevailing idea suggested.

The eyes, wanting in lustre, look out on the world around with a half-wistful yet resigned interest. No smile crosses the face when some circumstance rouses the youthful fellow-patients to laughter, and there is little increase of thoughtfulness when indications appear in the ward that something unusual is to take place. All seems to be noticed, and in a measure understood, but pleasure and pain are alike things of the past.

Very different is the expression in some diseases of the lung where there is considerable respiratory difficulty, such as chronic bronchitis and asthma. In these diseases the eyes, instead of being sadly thoughtful, may be full of apprehension or fear. The accompanying sketch from one of my note-books of a boy who suffered from asthma, has the following note by the side: "Boy of bronchitic or asthmatic aspect, cheeks fairly full, eyes staring, showing the white above and below iris. Mouth open, lines on either side of nose." The staring expression of the eyes is due to the widely opened eyelids which show, as the notes state, white above and below the iris. Another point mentioned in the notes which may be referred to in passing is fulness of the face. This is commonly present in chronic affections of the lung or pleura, and when the chest is stripped, the staring ribs and wasted arms may stand in strange contrast to the full face.

Another well-known facial expression not, however, peculiar to disease in children, is "the abdominal look." This expression may perhaps be said to be peculiar of itself, that is to say, it does not simulate any aspect of the face that may be produced by a condition of the mind. The abdominal look develops quickly in children suffering from summer diarrhœa. The sunken eyes and pinched features lead the mother to think the child has rapidly wasted: "He has wasted terribly during the last two days," is a common statement. Although in summer diarrhœa the loss of water may produce what is virtually wasting, the abdominal look probably owes little of its peculiar features to this form of wasting. A similar facial expression, it is needless to remark, may develop within a few minutes in a child as in an adult, as the result of an abdominal injury, in consequence of transfer of blood from the face to the veins of the abdomen, and it is no doubt rather to this collection of blood within the abdomen, than to loss of water, that the abdominal look at first makes its appearance in summer diarrhœa, though the loss of water cannot but be an important factor as the disease progresses.

A condition in which "the abdominal look" may appear without abdominal disease is pericarditis; but in bad cases of pericarditis, associated with this "look," instead of the comparative lethargy seen in cases of disease within the abdomen, there is great general distress. Possibly no disease occurring in a child past the age of infancy is so painful to witness as a bad case of pericarditis. The head thrown slightly back on the banked-up pillows, and the facial expression, possibly at first strike the observer less forcibly than the rapidly heaving chest, in which are noticed not only the hurried respirations, but the tremulous pulsations of the tumultuously acting heart. The agitated chest is a part of a seriously agitated body. A few restless movements of the arms may precede the flinging forward of the body on to the raised knees, in the hope of finding some relief in a new position. There is soon a falling back, however, to the pillows, when the half-closed lids may be wearily raised and the eyes turned to the observer with a look that seems to ask beseechingly yet hopelessly for aid.

Serious though the diseases usually are in which the abdominal look makes its appearance, sunken eyes, one of its most characteristic features, are also in less degree seen in what may be called delicate children. These children with partially sunken eyes, as a rule, are wanting in energy, and when kept standing for some time may faint. The hollow appearance of the eyes is no doubt due to similar physiological conditions to those present in cases of serious abdominal disease or injury; that is to say, a condition of the vaso-motor system exists which allows blood to collect in the abdomen which should circulate more freely through the skin and face. Curiously enough, children who present this sunken appearance of the eyes are not uncommonly said by their mothers to have swollen eyelids on rising in the morning. Sometimes swelling of the eyelids may still be present when children attend the out-patient department of a hospital. In a considerable percentage of these cases, examination of the urine shows the presence of cyclical albuminuria; that is to say, a trace of albumin is present in urine passed in the middle of the day, perhaps, also, in that of the evening, but not in the urine passed on rising in the morning. The albuminuria in such cases, with scarcely any doubt, is merely a consequence of the low vaso-motor tone, and does not indicate even slight disease of the kidneys.

Swelling of the eyelids in children may sometimes appear under apparently very slight exciting influences; for example, I have seen considerable swelling of the eyelids on one side of the face associated with earache on that side, and in a girl, very great swelling of the eyelids used frequently, but not always, to follow a warm bath. The mention of earache brings to mind the fact that in association with middle ear disease the facial aspect may sometimes be seriously altered by the presence of facial paralysis due to injury of the facial nerve in its course through the temporal bone. In such a case, it is scarcely necessary to remark, the forehead and eye, as well as the lower half of the face, suffer. In some cases of hemiplegia in children facial paralysis of the same extensive character occurs; that is to say, the paralysis does not affect, as in adults, only the face below the eye. This complete one-sidedness of paralysis is especially well seen in a baby or child when it cries, the comparative placidity of the diseased side being then very evident. In the accompanying sketch of the face of a baby affected with hemiplegia the facial paralysis is not absolute, but the comparative degrees of puckering of the forehead and of closure of the eyes on the two sides are clearly marked.

It is needless to say that the above cursory remarks merely briefly outline some of the facial appearances of ailing or sick children. Nothing has been said of the drowsy aspect of fever, of the fatuous face of chorea, or of the unintelligent expression associated with post-nasal adenoids. All reference to colour has also been omitted. The tints of anæmia, of empyema, the various conditions of jaundice, the colour of cardiac disease, would of themselves be sufficient to form the subject of an article. But although much has been omitted and much has been said which may be considered trivial, possibly some observations may have been made which are new to some readers and which may show that more may be learnt from the face in disease than at first appears.

## Figures and Tables

**Fig. 1. f1:**
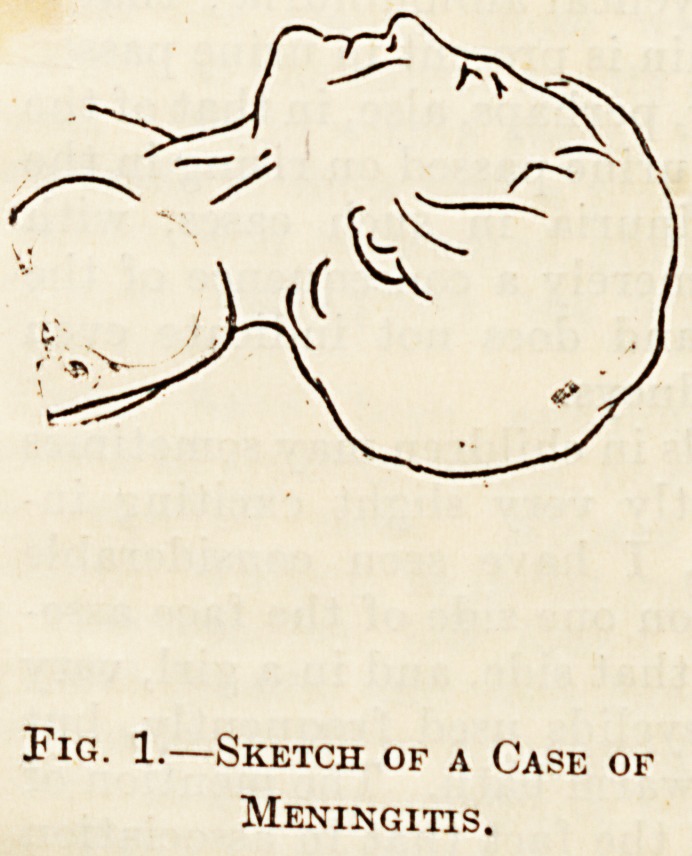


**Fig. 2. f2:**
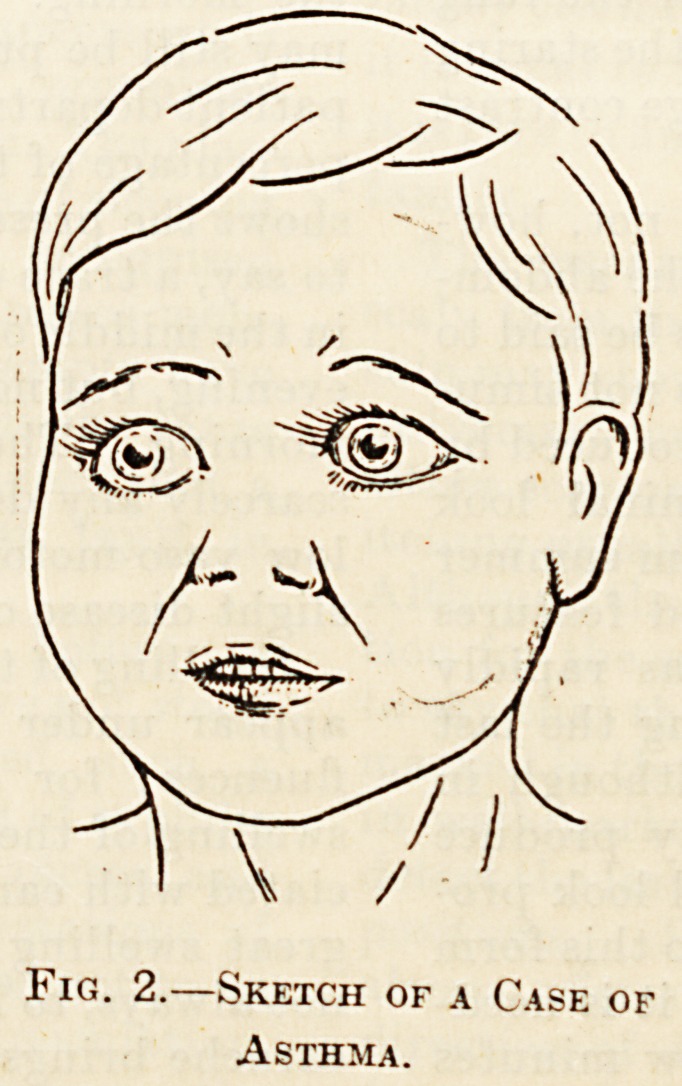


**Fig. 3. f3:**